# Label-free detection of creatinine using nitrogen-passivated fluorescent carbon dots†

**DOI:** 10.1039/d0ra06512a

**Published:** 2020-10-01

**Authors:** Shagun Kainth, Banibrata Maity, Soumen Basu

**Affiliations:** School of Chemistry and Biochemistry, Thapar Institute of Engineering and Technology Patiala 147004 India banibrata.maity@thapar.edu soumen.basu@thapar.edu; School of Chemistry and Biochemistry, Affiliate Faculty—TIET-Virginia Tech Center of Excellence in Emerging Materials, Thapar Institute of Engineering and Technology Patiala-147004 India

## Abstract

In the field of biochemistry and biosensing, the passivation of carbon dots using nitrogen dopants has attracted great attention, as this can control their photoluminescence (PL) properties and quantum yield. To date, in the fabrication of a sensing probe, the impact of the chemical composition of the passivating molecule remained unexplored. In this work, we chose a series of different nitrogen-rich precursors (such as urea, thiourea, cysteine, and glycine) and ascorbic acid to synthesize nitrogen-doped carbon dots (NCDs). A significant change in their surface states was obtained due to the evolution of variable contents of amino, pyridinic and pyrrolic nitrogen species, which is evident from X-ray photoelectron spectroscopy, and this leads to an increment in their PL quantum yields (PLQY ∼ 58%) and average lifetime values. Spectroscopic analysis revealed that a rise in the ratio of pyrrolic : amino groups on the surface of carbon dots cause a bathochromic shift and generate excitation-dependent properties of NCDs. Besides, these NCDs were used as fluorescence off–on sensing probes, where a PA-infested NCD solution was used to detect creatinine. Chiefly, fluorescence restoration was achieved due to the formation of Jaffe chromogen between creatinine and PA. However, all nitrogen-passivated carbon dot surfaces do not respond similarly towards creatinine and only non-amino-rich NCDs exhibit the maximum (50%) PL turn-on response. The PL turn-off–on methodology showed a satisfactory good linearity range between 0 and 150 μM with a detection limit of 0.021 nM for creatinine. Three input molecular logic gates were also designed based on the turn-off–on response of the NCDs@PA towards creatinine. Additionally, for analytical method validation, real-sample analysis was performed for creatinine, which showed good recoveries (93–102%) and verified that nitrogen passivation tailored the physicochemical properties and enhanced the sensing ability.

## Abstract 2

The role of passivation in CDs using different nitrogen precursors to evaluate its sensing proficiency towards creatinine quantification.

## Graphical abstract

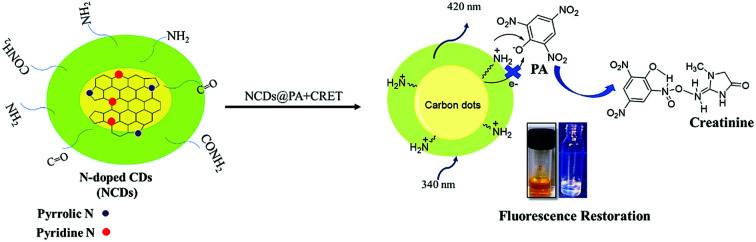

## 1. Introduction

Photoluminescent (PL) active materials have drawn great attention in the field of chemo/bio-sensing, bio-imaging and drug delivery. There are several varieties of classical fluorophore-like quantum dots (QDs) such as organic fluorophores that are used as analytical tools for toxic and important biological moieties.^1–3^ Nowadays, zero-dimensional carbon dots (CDs) have attracted as much attention as a novel fluorescent nanomaterial in the field of biosensing, energy storage, drug delivery, bio-imaging and catalysis. The CDs have gained the interest of researchers because of their several splendid properties such as tunable PL properties, multicolored emission, excellent chemical stability, low cost, facile preparation and ease of functionalization. The fundamental factor of CDs for exhibiting photoluminescence (PL) is the presence of quantum confinement and their surface groups,^2^ which are responsible for the variation in the bandgap, particle size as well as tunable emissive properties.

For the preparation of CDs, numerous traditional strategies such as strong acidic electrochemical oxidation, microwave-assisted synthesis, hydrothermal treatment and ultrasonic methods were reported in the literature.^4–7^ Even researchers were also succeeding towards various cost-effective amendments including the carbonization of various natural biomass materials such as milk, fruit juice, peels and coffee grounds used as carbon precursors to achieve different properties of CDs.^5,8–11^ The major components in CDs are carbon (C) and oxygen (O) showing blue fluorescence with a low quantum yield (PLQY), which often limit their applications and development.^12^ Thus, there is a need for surface modification or functionalization to enhance its PL property.

There were several parameters (such as synthetic routes, nature of precursors, and passivating substrate) that control the optical properties of CDs. The simplest way to tune the PL properties of CDs is *via* the surface state mechanism of interacting the carbon skeleton with different neighboring atoms using various passivating agents.^2,13–16^ Amongst the various passivating agents, the nitrogen precursor plays a crucial role as it adjusts easily to the carbon skeleton due to the comparable atomic size as carbon atoms.^17–19^ This creates multiple varieties of nitrogen species such as amino, pyrrolic, pyridinic and graphitic types in the structure of CDs, among which only the amino form is distributed on the surface and others at the center and edge of the graphene skeleton. Non-amino groups produce surface defects in the molecular core, which finally causes PL enhancement, whereas graphitic nitrogen modulates its color emitting properties. The development of these four different nitrogen species contributes to the difference in the optical properties. This eliminates or suppresses the original O-states in the CDs and facilitates the radiative recombination that induces high PLQY and tunable bandgap energy.^20,21^

In the literature, few synthetic routes were reported, in which different nitrogen-containing precursors were used to prepare NCDs. Ding *et al.* prepared multi-color NCDs using *p*-phenylenediamine and urea as precursors.^22^ They separated eight different types of NCDs by silica chromatography, which exhibited a variation in their emission wavelength in the range of blue to red emission (440–625 nm) and their PLQY values (8.5–35%).^22^ Many researchers also utilized various nitrogen-containing precursors such as ammonium citrate,^23^ a mixture of glucose and ammonium hydroxide,^24^ citric acid and branched polyethylenimine^24^ and ethylenediamine to prepare different NCDs. These different nitrogen-containing precursors cause quantum confinement and red-edge effect in the NCDs, which modulate their electronic properties. Apart from that, amino-rich precursors such as *o*-phenyl-diamine and 4-aminobutyric acid were also reported for yellow-emissive NCDs, which was implemented to detect fluoroquinolone derivatives *via* a fluorescence sensing approach.^25^ Likewise, Song *et al.* explored dual-emissive NCDs using a single nitrogen precursor (a mixture of *o*-phenyl-diamine and *ortho*-phosphoric acid) for the intercellular detection of lysine.^26^ Many other nitrogen sources such as 1,2,4-triaminobenzene were also utilized to prepare NCDs, which were used for the cell imaging and bifunctional sensing.^27^ These studies indicate that passivation of CDs using different nitrogen precursors is the simplest route to obtain long-emissive-range CDs with high PLQY, which is essentially required to enhance the reliability of the nanoprobe towards different toxic moieties.^28,29^

Among the various toxin moieties, 2,4,6-trinitrophenol (picric acid, PA) was found to be explosive due to the existence of electrophilic nitro groups and high water solubility. Immense evidence for its quantification was known with hydrophilic and hydrophobic CDs.^30,31^ Due to the excited-state electron transfer/charge transfer interaction, CDs exhibit high PL response (significant quenching) for PA.^31,32^ Still, in Jaffe's reaction, PA is used for the determination of creatinine. Many sensing strategies were also reported for the detection of creatinine. Parmar and co-workers followed a colorimetry-based evaluation of creatinine using picric acid–capped silver nanoparticles.^33^ A gluten-stabilized fluorescent gold quantum cluster was used as a fluorophore to quantify the amount of creatinine in the blood sample.^34^ With the change in creatinine concentration level in blood and urine (37–250 mg dl^−1^), it causes renal diseases, muscular dystrophy and myasthenia.^34,35^ Until now, the aforementioned methods have certain limitations w.r.t. their experimental method, cost-effectiveness, selectivity and sensitivity towards creatinine. Therefore, monitoring of creatinine in biological samples of patients is becoming a major concern in these days. In this context, to the best of our knowledge, no work has been reported where the effect of different nitrogen precursors on the CD sensing ability was explored for the quantification of creatinine.

Herein, we examined the impact of different nitrogen precursors such as urea, thiourea, cysteine and glycine as passivating agents on the photoluminescence and photophysical properties of NCDs. We also assessed the role of surface passivation on the sensing efficacy of NCDs towards biomolecules. Besides, implementation of different interconnecting molecular logic gates (IMP, AND, and OR) was framed to design a nano-senor device.

## 2. Experimental section

### 2.1 Materials and characterization techniques

Thiourea, urea, cysteine, glycine and ascorbic acid were purchased from Loba Chemie Pvt Ltd (spectrograde), India. All these reagents were used directly by preparing their aqueous solution using distilled water (18.2 MΩ cm, Millipore). The NCDs were synthesized using a Multiwave-300 microwave synthesis reactor (Anton Paar, USA). A LS-55 spectro-fluorophotometer (PerkinElmer) was used to study its optoelectronic properties. The fluorescence lifetime for NCDs in the presence and absence of the analyte was well determined by time-correlated single-photon counting (TCSPC) measurements using a delta-flex modular fluorescence lifetime system (HORIBA Scientific) equipped with a light-emitting diode (LED) light source (340 nm) with an instrument response function (IRF) of ∼200 ps. The synthesized NCDs were also characterized using a high-resolution transmission electron microscope (HRTEM) (TALOS F200S G2, 200 KV, FEG, CMOS Camera 4 K × 4 K, In Column EDS detectors) for the analysis of their surface morphology and particle size using a drop-casting technique. Moreover, Fourier transform infrared spectroscopy-attenuated total reflectance (FTIR-ATR, Shimadzu QATR-S model), XPS, X-ray photoelectron microscopy and energy-dispersive X-ray (EDX) spectroscopy techniques were carried out to gain better insights into the NCD surface.

### 2.2 Synthesis of NCDs

The microwave-assisted synthesis was followed to obtain nitrogen-doped carbon dots (NCDs) using different nitrogen precursors such as urea, thiourea, glycine, and cysteine and carbon source such as ascorbic acid, which are abbreviated as U-NCDs, T-NCDs, G-NCDs and C-NCDs respectively. First, ascorbic acid and the respective nitrogen-containing precursors (1 : 1 w/w) were dissolved in 10 ml of distilled water and kept for 5 min at 130 °C in a microwave synthesizer. Then, the obtained yellowish-brown solution was purified by centrifugation at 5000 rpm and the supernatant was passed 3–6 times through filter membranes of 0.22 μm to obtain pure NCDs, and their PL properties were recorded at *λ*_ex_ = 340 nm.

### 2.3 Experimental procedure for fluorescence quenching of NCDs and PA

We analyzed the fluorescence emission spectra of all the synthesized NCDs (U-NCDs, G-NCDs, T-NCDs and C-NCDs) using an LS-50 PerkinElmer spectrofluorometer. First, 100 μl of NCDs from the prepared stock solution was pipetted out in a 2 ml cuvette, and the rest of the volume was made up with distilled water and their emission spectra were recorded at *λ*_ex_ = 340 nm. To the same cuvette containing NCDs, the respective amount of PA solution was gradually added from the prepared stock solution and their emission spectra were recorded. The addition of PA solution was continued till the maximum fluorescence quenching was achieved.

### 2.4 Determination of PLQY

The PLQY of all four NCDs was evaluated using eqn (1), for which quinine sulphate (*ϕ*_r_ = 0.546)^36^ dissolved in 0.01 M H_2_SO_4_ was used as the reference solution:

1 *φ* = *φ*_r_ × (*I*/*I*_r_) × (*A*_r_/*A*) × (*η*^2^/*η*_r_^2^)

where ‘*φ*’ and ‘*φ*_r_’ denote the PLQY of the sample and reference respectively, ‘*I*’ the integrated area of fluorescence intensity, ‘*A*’ the absorbance value and ‘*η*’ the refractive index.

### 2.5 Determination of quenching constant and limit of detection (LOD)

The quenching constant of different NCDs in the presence of PA was evaluated using the following Stern–Volmer equation:2
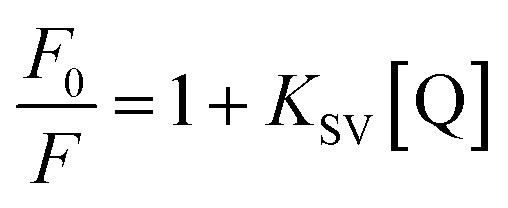


The parameters, namely, *F*_0_ and *F* represent the fluorescence intensity in the absence and presence of PA, respectively. *Q* is the concentration of PA. *K*_SV_ represents the Stern–Volmer quenching constant. The determination of the *K*_SV_ value was done from the slope obtained from the plot of *F*_0_/*F vs.* [Q].

### 2.6 Determination of the binding constant

The interaction studies between NCDs with PA were determined using the 1 : 1 stoichiometry of the Benesi–Hildebrand equation as follows:^37^3
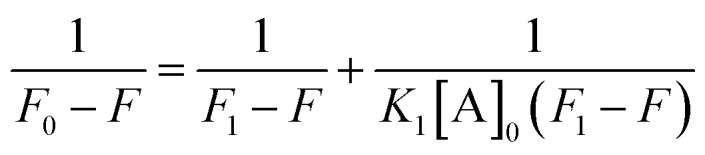
where *F*_0_ and *F* are the fluorescence intensities of NCDs in the absence and presence of PA. *F*_1_ is the fluorescence intensity for the 1 : 1 complex. Therefore, the B–H plot of 1/(*F*_0_ − *F*) *vs.* 1/[A]_0_ should give a straight line. From the slope and intercept of the line, the binding constant (*K*_1_) values for different NCDs have been estimated.

### 2.7 Experimental procedure to detect creatinine based on fluorescence restoration

The fluorescence of the prepared NCDs@PA systems was explored for the quantification of creatinine (CRET). The efficacy of this nanoprobe was evaluated based on ligand replacement and fluorescence restoration approach (turn-on). To the NCDs@PA system, 1 mM stock solution of CRET was gradually added and the respective fluorescence spectra were recorded at the same excitation wavelength (*λ*_ex_ = 340 nm). The addition of CRET results in the fluorescence enhancement of NCDs@PA from which we have further quantified its detection limit using the following equation:4
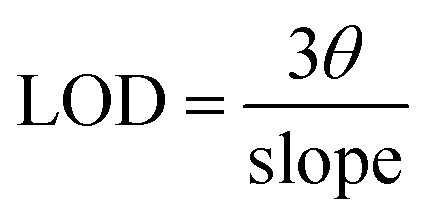
where ‘*θ*’ is the standard deviation and slope obtained from the respective plot.

### 2.8 Sensing of creatinine detection in urine

The urine sample was collected from laboratory co-workers, stored at 4 °C and discarded after 24 h. The urine samples were centrifuged at 4000 rpm for 30 min and filtered using a syringe filter (0.44 μm). Aliquots of the urine samples (3 ml) were treated similarly to calibration standards and the fluorescent intensity at 450 nm was monitored.

## 3. Results and discussions

### 3.1 Size and surface composition of different NCDs

**Fig. 1:**
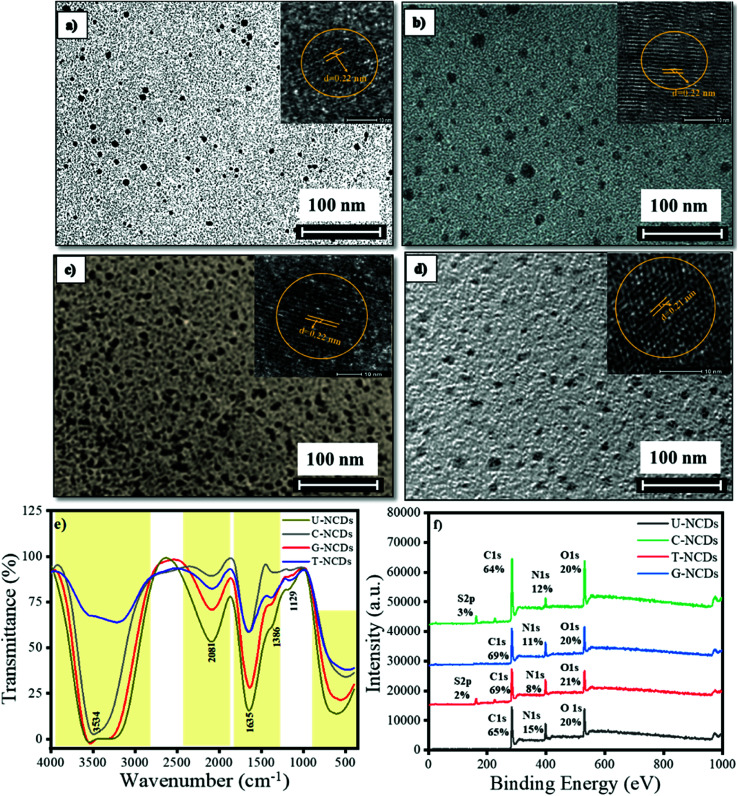
Particle size, surface compositions and elements: (a) U-NCDs, (b) C-NCDs, (c) G-NCDs, (d) T-NCDs, (e) FTIR, and (f) XPS spectra.

The size and morphology for different NCDs were characterized by HRTEM analysis. The HRTEM images indicated that all the NCDs were spherical and monodispersed with a diameter range of 5–8 nm (Fig. 1 and S1a†). The lattice fringes of all four NCDs showed a spacing of 0.22 nm (Fig. 1) corresponding to the (100) in-plane lattice fringe of graphene, which justified that the sp^2^ carbon skeleton was present in NCDs.^38^ The surface composition for all four NCDs was assessed by FTIR spectroscopy, as shown in Fig. 1e. In the FTIR spectra, the intense band appeared at 3583 cm^−1^, which is assigned to the N–H stretching vibrations (pyrrolic nitrogen). The band at 2081 cm^−1^ is assigned to the N–H stretching vibration and the band at 1635 cm^−1^ to pyrrolic, pyridinic, and chemisorbed nitrogen.^39^ The other bands appearing at 1386 and 1129 cm^−1^ were assigned to the C–N (graphitic nitrogen) and C–O stretching vibrations.^39,40^ The attained bands revealed that all four NCDs were functionalized with variant hydrophilic groups, which give the primary idea to understand the interactions between NCDs and PA. The surface states and composition in NCDs were also characterized by XPS. The full spectra are shown in Fig. 1f, which reveal that all four NCDs were composed of the same elements C, N, and O having different ratios, whereas C-NCDs and T-NCDs also have a sulfur peak. The C 1s band de-convoluted into three peaks at 284.8, 286.1, and 288.5 eV, which are assigned to C=C/C–C, C–N/C–O and C=O/–CONH– respectively (ESI, Fig. S1a† and Table 1). The N 1s band was also split into three peaks, corresponding to pyridinic N (398.4 eV), amino N (399.2 eV) and pyrrolic N (400.2 eV) as mentioned in Table 2 and Fig. S2.† In the O 1s spectrum, two peaks were found at 531.2 and 533.0 eV, revealing the existence of C–O and C=O (Fig. S2† and Table 3). The contribution of each of the several surface groups was calculated from their de-convoluted spectra. The insight evaluation revealed that the content of C=O/–CONH– surface groups (Tables 1 and 3) exists in the order of U-NCDs > C-NCDs ∼ G-NCDs > T-NCDs, whereas from Table 2, the content of amino groups exists in the order of T-NCDs > C-NCDs ∼ G-NCDs > U-NCDs. This specifies that different nitrogen sources generate different ratios of nitrogen surface groups and different degrees of oxidation, which cause a red-shift in the emissive spectra of NCDs (detailed in Section 3.2)^40^ and can modulate and improve their selectivity towards toxin biomolecules.

**Table 1:** XPS data analysis of C 1s spectra of different NCDs

NCDs	U-NCDs	C-NCDs	G-NCDs	T-NCDs
C–C/C=C	60%	41%	40%	65%
C–N/C–O	22%	42%	45%	21%
C=O/–CONH–	18%	15%	14%	13%

**Table 2:** XPS data analysis of the N 1s spectra of different NCDs

NCDs	U-NCDs	C-NCDs	G-NCDs	T-NCDs
Pyridinic N	15%	6%	10%	5%
Amino N	—	13%	20%	41%
Pyrrolic N	85%	79%	69%	53%

**Table 3:** XPS data analysis of O 1s spectra of different NCDs

NCDs	U-NCDs	C-NCDs	G-NCDs	T-NCDs
C–O	40%	43%	49%	53%
C=O	60%	57%	51%	47%

### 3.2 Optical properties of different NCDs

**Fig. 2:**
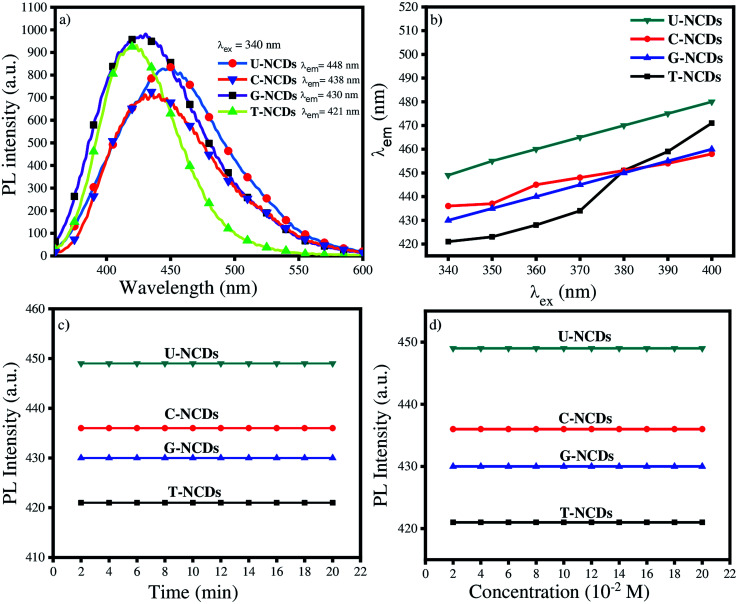
Fluorescence studies of NCDs: (a) emission properties of different NCDs at *λ*_ex_ = 340 nm, (b) emission behaviour at different excitation wavelengths, (c) impact of UV irradiation and (d) different ionic strengths of NaCl on their PL properties.

UV-Vis absorption spectroscopy and the photoluminescence (PL) spectra were recorded to investigate the optical properties of NCDs. UV-Vis spectra showed two common major absorption bands at 235 nm and 270 nm respectively for the four different NCDs, as shown in Fig. S1b,†*i.e.* ascribed to the π–π* transition of the sp^2^ C=C conjugated system and the n–π* transition of the C=O groups on the surface.^41^ The PL spectra of all the NCDs were assessed at *λ*_ex_ = 340 nm. The experimental results indicated that the emission wavelength lies in the range from 425 to 450 nm, whereas in several reports oxygen-rich CDs have emission at 420 nm.^42^ The results indicated that nitrogen passivation leads to a bathochromic shift in the emission spectra of NCDs. The descending order of the bathochromic shift for NCDs is as follows: U-NCDs > C-NCDs > G-NCDs > T-NCDs (Fig. 2a). This trend occurred due to the different nitrogen contents (as mentioned in Table 2) and deformation in the plane that decreases their bandgap energy, which was caused by various surface groups such as –CONH_2_, and different nitrogen species (pyridinic, pyrrolic and amino).^2^

However, the emission spectra of NCDs were also recorded at different excitation wavelengths that range from 340 to 390 nm (Fig. 2b and S3†). The experimental studies revealed that the PL peak position undergoes a bathochromic shift in their emission wavelength from 400 to 460 nm dependent on the excitation wavelength. This feature of NCDs arises mainly due to the presence of various surface states such as C–H, C=O, C–NH_2_ and C–OH (supported by XPS and FTIR-ATR analysis).^41,43,44^

PLQY is also one of the most inherent fluorescence properties of a fluorophore. Therefore, we calculated the PLQY values using quinine sulphate as a reference (*ϕ*_r_ = 0.546) in eqn (1) (Section 2.4) for U-NCDs, C-NCDs, G-NCDs and T-NCDs, which are found to be 58%, 51%, 51% and 31% respectively due to the higher nitrogen content in the carbon core and pyrrolic structure that effectively improves the PLQY (evaluated in XPS analysis). These results were justified from previous reports where hetero-atoms and surface passivation can enhance the PLQY of CDs w.r.t. pure carbon- and oxygen-rich CDs.^2,40,45,46^

The photostability of NCDs was also assessed from the PL studies under UV irradiation and at different ionic strengths of the NaCl salt. The studies revealed that after exposure to continuous UV irradiation for 20 min, the emission spectra were not disturbed, which signified their good anti-photobleaching property. Even in the presence of different ionic strengths (NaCl), their emission peak did not get disturbed, which guarantees their sensing applicability in the biological and aquatic systems (Fig. 2c and d).

### 3.3 Effect of pH on different NCDs

The effect of pH plays a vital role in controlling the sensing efficacy of probe towards biomolecules and metal ions. It was found that the PL intensity and emission wavelength of NCDs remain constant with change in the pH values (Fig. S4†). This result reflects that protonation and deprotonation have minimal impacts on the electronic transition of NCDs. Therefore, it can be demonstrated that NCDs are good potential candidates for use in biological systems.^47^

### 3.4 Interaction studies of different NCDs with PA

**Fig. 3:**
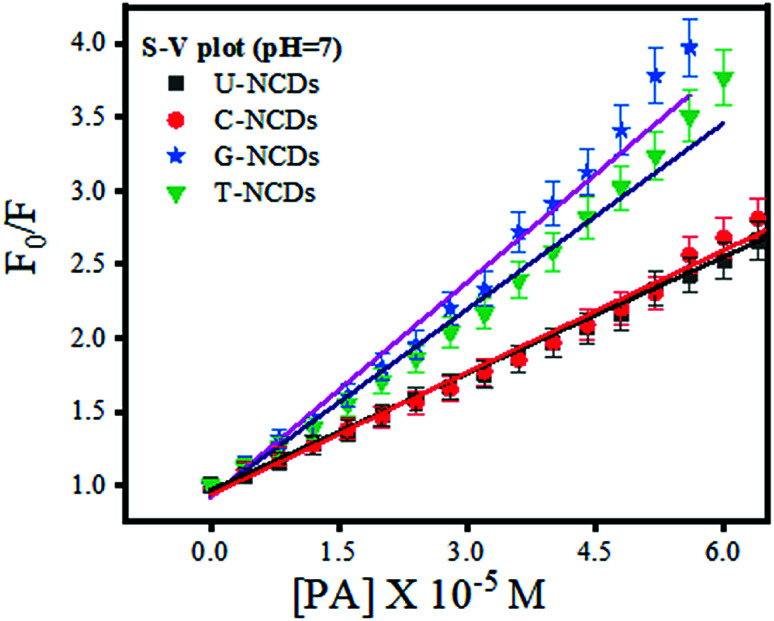
Stern–Volmer plot for U-NCDs, C-NCDs, G-NCDs and T-NCDs at pH = 7 in the presence of PA.

We performed the fluorescence quenching study to explore the interaction of PA with four different NCDs. The experiment showed a remarkable reduction in the PL intensity among all NCDs on the addition of PA. This observed phenomenon occurred due to the excited-state electron-transfer from electron-rich species on the NCD surface to the electron-deficient PA.^44^ Moreover, to evaluate its quenching efficacy, the Stern–Volmer quenching experiment was performed to calculate Stern–Volmer quenching constant values (*K*_SV_) at three different pH values of ∼4, 7 and 9 (Fig. 3 and S5†) using eqn (2). The experimental results indicated that all four NCDs showed a higher rate of quenching constant at pH = 7 w.r.t. other pH values. This observed behavior is due to the formation of anion–cation pairs *via* strong electrostatic interactions between the nitrogen-rich surface of NCDs and PA and the absence of deprotonation and protonation of PA, which frequently occurs at lower and higher pH values.^48^ Additionally, the experiment studies revealed that among all four NCDs, U-NCDs and C-NCDs showed similar quenching efficiencies, with *K*_SV_ ∼ 0.27 × 10^5^ M^−1^ whereas, for T-NCDs, G-NCDs have similar *K*_SV_ ∼ 0.42 × 10^5^ M^−1^ values. The evaluated result revealed that all four NCDs have inconstant electrostatic interactions (formation of anion and cation pairs) with PA.

**Fig. 4:**
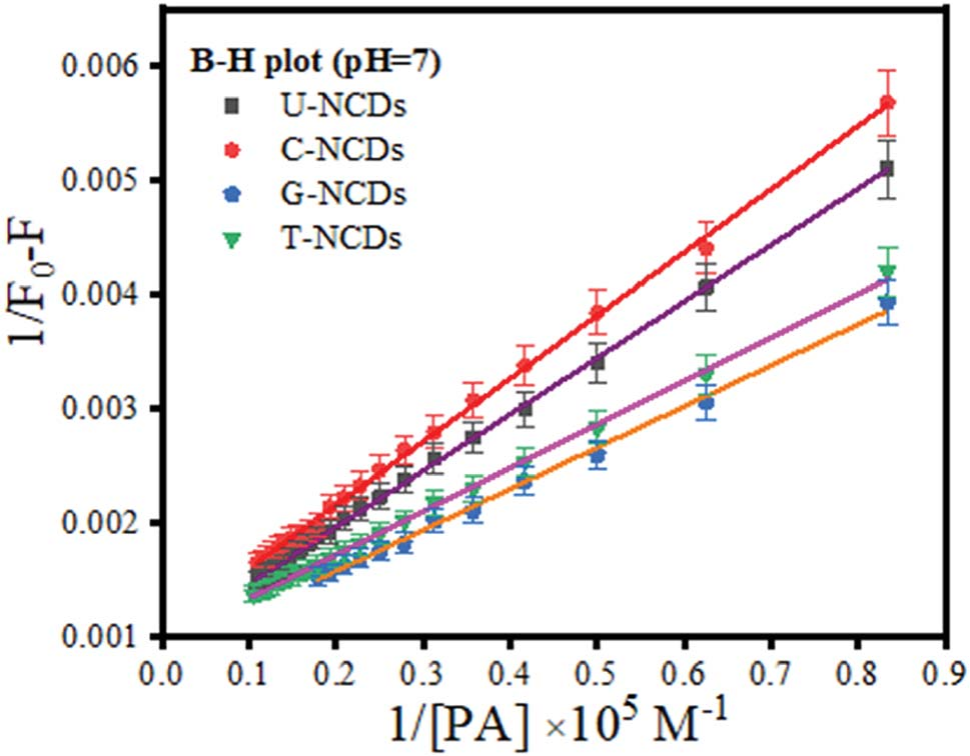
B–H plot for U-NCDs, C-NCDs, G-NCDs and T-NCDs at pH = 7 in the presence of PA.

Subsequently, to confirm the binding efficacy for PA, the B–H plot and binding constant were evaluated (using eqn (3)). The experimental studies revealed that at pH = 7, all four NCDs exhibited excellent binding at pH = 4 and 9 with a binding constant of 1.79 × 10^7^, 2 × 10^7^, 2.66 × 10^7^ and 2.85 × 10^7^ M^−1^ for U-NCDs, C-NCDs G-NCDs and T-NCDs respectively (as shown in Fig. 4 and S6†). This apparent increase in this binding and quenching efficacy from U-NCDs to T-NCDs towards PA occurs due to the rise in the content ratio of amino surface groups that enhance the formation of anion and cation pairs with PA, which causes energy transfer process; similar results were also reported in literature.^30,44,48^ Therefore, nitrogen passivation can be a superlative route to control the selectivity of the probe to sense different biomolecules.

### 3.5 Establishment of the PL sensing method for creatinine

**Fig. 5:**
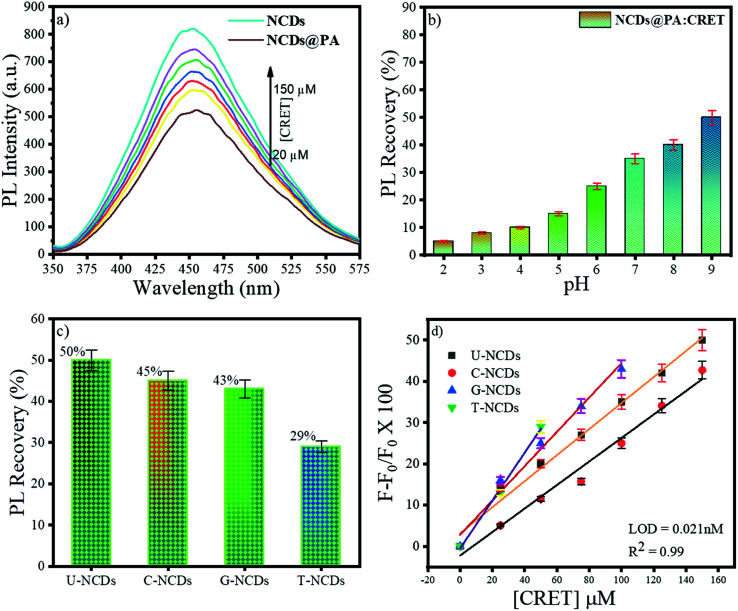
Fluorescence behaviour of NCDs@PA in the presence of CRET at pH = 9; (a) impact of different concentrations of CRET on the PL intensity, (b) effect of pH on the PL recovery of NCDs in the presence of PA, (c) % PL recovery of different NCDs@PAs and, (d) plot *F* − *F*_0_/*F*_0_*vs.* [CRET].

**Scheme 1:**
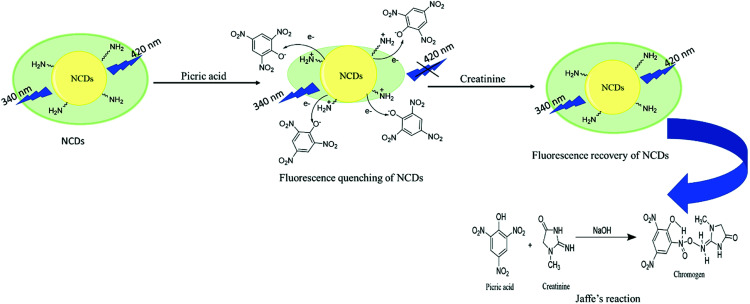
Turn-off–on mechanism of NCDs@PA in the presence of CRET.

The fluorescence restoration method was followed by using NCDs@PA for the CRET quantification. First, the pH of the system was adjusted to a mild basic state by Tris HCl buffer (0.001 M) to study the interaction between CRET and NCDs@PA. It showed rapid fluorescence recovery due to the strong complexation process between PA with CRET, and the color of the solution changed from yellow to orange-red, which confirmed the formation of red Jaffe chromogen (inset of Fig. 5b).^49^ The variable fluorescence recovery was also attained for NCDs, and U-NCDs@PA exhibited maximum PL restoration (50%), whereas T-NCDs@PA exhibited minimum PL restoration (29%) in the presence of CRET (Fig. 5c). These discrepancies in the phenomenon were observed due to the difference in the ratio of amino groups on its surface (Tables 1 and 2), as it enhances its association with PA *via* hydrogen bonding, which causes hindrance in the PA and CRET complex formation (shown in Scheme 1).

Furthermore, NCDs@PA linear response was recorded by successive addition of CRET at different concentrations ranging from 0 to 150 μM (Fig. 5d) for its quantification. The results indicated a good linear relationship between the PL signal ranging from 0 to 150 μM with a detection limit of 0.021 nM calculated using eqn (4). These results symbolized that a less amino-rich NCDs@PA system can be used as an effective colorimetric as well as fluorometric device for creatinine detection.

### 3.6 Evaluation of NCDs@PA sensing efficacy towards creatinine

The selectivity and sensitivity of the prepared nanosensor were validated through interference studies. Several relevant biological molecules such as fructose (Frct), glucose (Glu), galactose (Gla), cysteine (Cys), glutathione (GSH), and glycine (Gys) and metal ions such as Fe^3+^, Mg^2+^, Zn^2+^, Ca^2+^, and citrate were chosen to study the efficacy of the NCDs@PA system by maintaining their concentration. The evaluated result indicates that foreign moieties showed no significant interaction with the NCDs@PA system and does not cause any hindrance in its PL recovery for creatinine (Fig. S7†).

Furthermore, the effectiveness of U-NCDs@PA was justified by comparing some current methods and detecting probes mentioned in Table 4. These studies indicate that our prepared material shows utmost sensing similarities in comparison to colorimetric and supramolecular materials. Moreover, NCDs@PA probe was found to be eco-friendly, as no hazardous material was used in their preparation method.

**Table 4:** Comparison of the efficacies of various probes with that of NCDs@PA towards CRET

Methods	Detection system	LOD	Linearity	Reference
Colorimetry	Au@BSA	8.4 nM	0.01–1 μM	50
Colorimetry (paper-based sensor)	Jaffe reaction	72 nM	0.1–30 μM	51
Amperometry	—	0.0003 nM	1–150 μM	52
Fluorometry	1,8-Napthalimide (FCD–Pd complex)	0.0003 nM	—	53
Fluorometry	Gluten-stabilized fluorescent gold quantum cluster	2 nM	20–250 μM	54
Fluorometry	Supramolecular metallic ensemble in H_2_O : DMSO (6 : 4 v/v)	10 pM	—	55
Fluorometry	N-Doped carbon dots (H_2_O)	0.021 nM	0–150 μM	Current work

### 3.7 Time-resolved PL measurements of NCDs in the presence of PA and creatinine

**Fig. 6:**
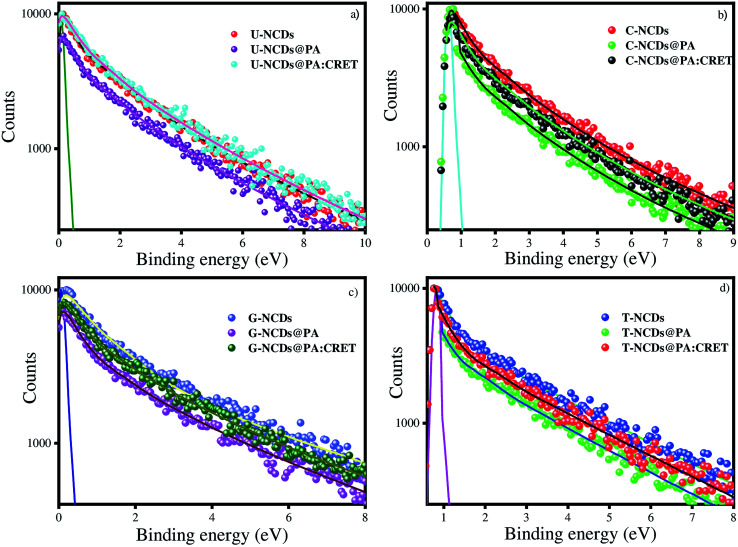
Time-resolved spectra for (a) U-NCDs, (b) C-NCDs, (c) G-NCDs and (d) T-NCDs in the absence and presence of PA and CRET at pH = 9.

Time-resolved measurement was recorded for NCDs at their respective emission wavelengths by exciting the samples at *λ*_ex_ = 340 nm (Fig. 6). The average PL lifetime was found in the range of 1–2 ns, which is very close to the previously reported values.^56^ Further to validate the interaction of PA with different NCDs, the lifetime decay studies were recorded in the presence and absence of PA (Fig. 6). The outcomes displayed a significant decline in its average lifetime values upon the addition of the PA solution (21 mM, 150 μl) and showed dynamic quenching characteristics (Fig. 6 and Table 5). Moreover, from these average lifetimes and PLQY values of NCDs, radiative (*k*_r_) or non-radiative (*k*_nr_) decay rate was calculated using the following equations:5
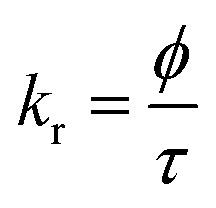
6
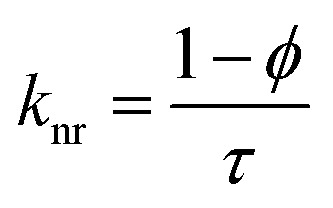
where *ϕ* is the quantum yield and *τ*_av_ is the average lifetime of the respective systems. Further, it is evident from Table 5 that in the presence of PA, the non-radiative decay rate (*k*_nr_) gets enhanced w.r.t. radiative decay (*k*_r_) for NCDs, which confirms that PA significantly quenched its fluorescence. Additionally, the rate of electron transfer from NCDs to PA and PA + CRET has also been calculated using the following equation:7
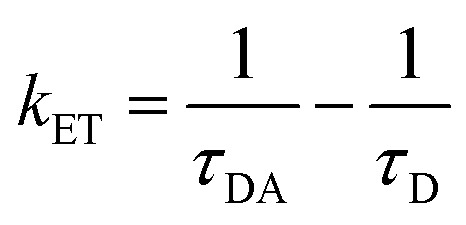
where, *τ*_D_ and *τ*_DA_ represent the average lifetime values of NCDs (donor) and NCDs + PA (acceptor) respectively. It was found that the rate of electron transfer became maximum for T-NCDs (∼0.76 ns), whereas minimum for U-NCDs (∼0.1 ns) in the presence of PA. This difference in the *k*_ET_ values arises due to the ratio of the amino groups on its surface as found from XPS analysis. Moreover, in the presence of CRET, the energy transfer process gets declined for the NCDs@PA system, which justifies that the fluorescence restoration mechanism takes place (Scheme 1) due to the formation of the picric–creatinine complex. Here, *τ*_D_ and *τ*_DA_ are considered as NCDs + PA and NCDs + PA + CRET respectively.

**Table 5:** Photophysical parameters of NCDs in the presence and absence of PA and CRET

Systems	*Φ*	*τ*_av_ (ns)	*k*_r_ (× 10^9^ s^−1^)	*k*_nr_ (× 10^9^ s^−1^)	*k*_ET_ (× 10^9^ s^−1^)
U-NCDs	0.58	2.12	0.27	0.23	—
U-NCDs + PA	0.25	1.74	0.17	0.4	0.1
U-NCDs + PA + CRET	0.48	1.9	0.25	0.27	0.05
C-NCDs	0.51	1.63	0.31	0.34	—
C-NCDs + PA	0.25	1.33	0.24	0.58	0.14
C-NCDs + PA + CRET	0.38	1.51	0.25	0.41	0.05
G-NCDs	0.51	1.71	0.31	0.31	—
G-NCDs + PA	0.22	1.39	0.18	0.52	0.13
G-NCDs + PA + CRET	0.39	1.55	0.25	0.39	0.06
T-NCDs	0.31	1.08	0.37	0.56	—
T-NCDs + PA	0.20	0.59	0.25	1.44	0.76
T-NCDs + PA + CRET	0.26	0.97	0.26	0.77	0.1

### 3.8 Strategy to design molecular logic gates

**Scheme 2:**
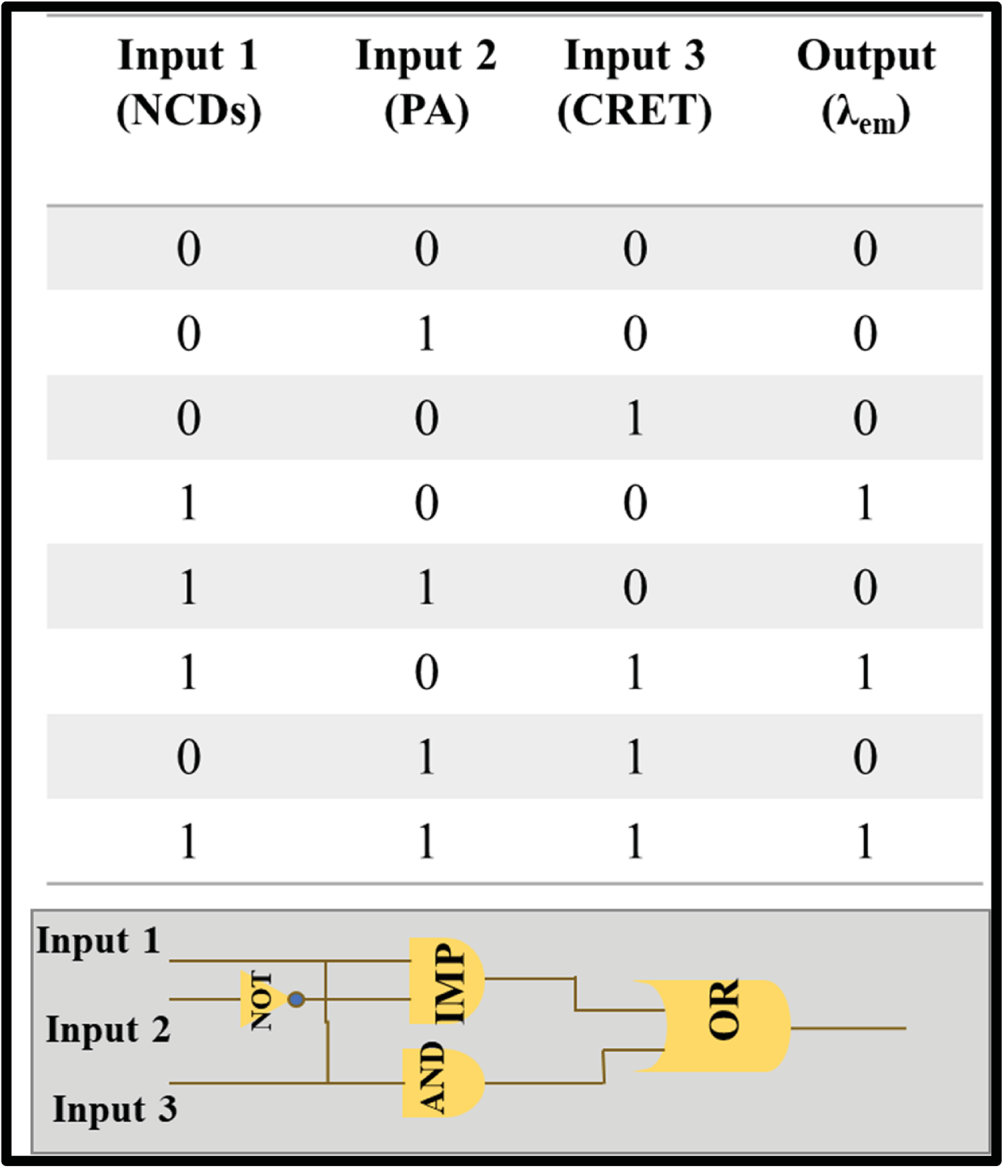
Description of truth table and design of the molecular logic gate.

Molecular logic gates were also designed with three inputs based on the turn-off–on response of NCDs towards creatinine in the presence of PA at a constant concentration (21 mM, 150 μl). We define “0” and “1” as binary inputs of the logic gates for the presence and absence of analytes, respectively. The output signal of the logic gate depends on the PL intensity of NCDs in which “0” refers to fluorescence quenching and “1” for non-quenching fluorescence. Three different IMP, OR and AND gates were evaluated for the relation of the following PL behavior for NCDs with PA and creatinine respectively. The results specify that the combination of only three inputs, namely, NCDs (100), NCDs/creatinine (101) and NCDs/PA/creatinine (111), was found to be suitable to validate the aforementioned fluorescence enhancement (output = 1) (Scheme 2). Therefore, this system can be used as a portable device simulating the fluorescence-induced signals into logic gate models.

### 3.9 Sensing mechanism between NCDs@ PA and creatinine

The prepared probe NCDs@PA exhibited high selectivity and sensitivity towards creatinine. To understand the sensing mechanism between NCDs@PA and creatinine, UV-Vis absorption and fluorescence emission studies were employed. In Fig. S8a,† the absorption spectrum of PA and fluorescence emission spectra of NCDs exhibited a wide overlapping region, which suggested that the fluorescence quenching of NCDs in the presence of PA was due to fluorescence resonance energy transfer (FRET) or the inner filter effect. Fan *et al.* obtained similar results with PA, and their experimental reports revealed that due to the inner filter effect between PA and CDs, the fluorescence quenching took place in CDs.^44^ However, in our case, time-resolved emission studies suggested that the average lifetime values of all NCDs@PA significantly declined (Table 5). Thus, it justified that the NCDs quenching phenomenon is due to FRET mechanism. The FRET mechanism can be explained based on the abundance of different nitrogen species (pyridinic-N, pyrrolic-N, and amino groups) on the surface of NCDs (Table 2), which can effectively bind with PA through acid–base pairing.^57^ Further to validate the sensing mechanism between NCDs@PA and creatinine, we have recorded the UV-Vis spectra of NCDs, NCDs@PA and NCDs@PA@CRET (Fig. S8b†). It was found that the addition of creatinine to the NCDs@PA system results in a new absorption band at 236 nm, which was assigned due to Jaffe complexation.^58^ Moreover, in the presence of creatinine, the average lifetime values of NCDs@PA further enhanced (mentioned in Table 5). Therefore, it can be concluded that the Jaffe complex formation reduced the FRET phenomenon, which clarifies that restoration of fluorescence in NCDs is the fundamental origin for the detection of creatinine.

### 3.10 Real sample analysis for method validation

To validate the proposed method for the detection of creatinine, fluorescence recovery analyses were performed with the real system. The urine samples were spiked with a suitable amount of standard creatinine solution. The probe showed a similar response as the prepared standards with 99% recovery determined using a calibration curve mentioned in Table 6. These findings suggested that the developed methodology can be used as a selective tool to detect creatinine in real samples.

**Table 6:** Sensing efficiency of NCDs@PA towards creatinine in the real sample

Spiked samples	Creatinine added (μM)	Creatinine found (μM)	Recovery (%)
1	0	0	0
2	2	1.85	93
3	50	48	96
4	80	82	102
5	115	114.5	99

## 4. Conclusions

In summary, different nitrogen-containing precursors have been used to synthesize different types of NCDs and their impacts on the photophysical properties have been explored. The designated synthetic parameters and the nitrogen-rich passivating agent were found to be helpful in shaping their surface properties demonstrated by FTIR-ATR spectroscopy and XPS analysis. These nitrogen precursor-dependent tailoring of the surface properties of NCDs established a red-shift in the fluorescence emission spectra, high PLQY and average lifetime values. Another most observable significance of different surface states was the modulation in their binding efficacy towards picric acid. The effective binding of four NCDs with PA was only achieved at pH = 7 due to the fast energy transfer process and acid–base pair formation, while no such response was obtained with creatinine. Despite this fact, the PA-infested NCD solution (pH = 9) was used to determine creatinine based on the regaining fluorescence efficacy in the same pot. The studies indicated that the U-NCDs@PA system accomplishes a prominent response in the presence of creatinine with 50% PL recovery and the LOD is 0.21 nM. Correspondingly, PL studies were supported with time-resolved emission measurements of NCDs, which verified the turn-off–on response of NCDs in the presence of PA and creatinine. Furthermore, the proposed nanosensor (U-NCDs@PA) was applied for creatinine determination in urine samples, which showed appreciable recoveries (93–102%). Subsequently, based on the coordination and ligand replacement reaction between NCDs, PA and creatinine, three-input molecular logic gates were designed. Overall, this nitrogen doping in CDs was found to be a simple and ecofriendly route to design an on-site creatinine detection system for biomedical applications.

## Ethical statement

All real sample analysis was performed in accordance with the institutional guidelines and with the consent of the participant (who is one of the co-author of this paper) at Thapar Institute of Engineering and Technology.

## Conflicts of interest

The authors have no conflict of interest in the publication of this manuscript.

## Supplementary Material

RA-010-D0RA06512A-s001
