# Supplementary material for: Label-free detection of creatinine using nitrogen-passivated fluorescent carbon dots
Source: RSC Adv. 2020 Oct 1;10(60):36253–64. doi: 10.1039/d0ra06512a (PMC9056981; doi:10.1039/d0ra06512a)
Supplement: RA-010-D0RA06512A-s001 [file RA-010-D0RA06512A-s001.pdf]

Electronic Supplementary Material (ESI) for RSC Advances.
This journal is © The Royal Society of Chemistry 2020

Supporting Information

Label-Free Detection of Creatinine by Nitrogen Passivated Fluorescent Carbon-dots

Shagun Kainth[1], Banibrata Maity[1,2*] and Soumen Basu[1,2*]

[1]*School of Chemistry and Biochemistry, Thapar Institute of Engineering and Technology Patiala 147004, India*

[2]*School of Chemistry and Biochemistry, Affiliate Faculty—TIET-Virginia Tech Center of Excellence in Emerging Materials, Thapar Institute of Engineering and Technology, Patiala-147004, India*

**Corresponding Author E-mail: banibrata.maity@thapar.edu (B. Maity); soumen.basu@thapar.edu (S. Basu)*

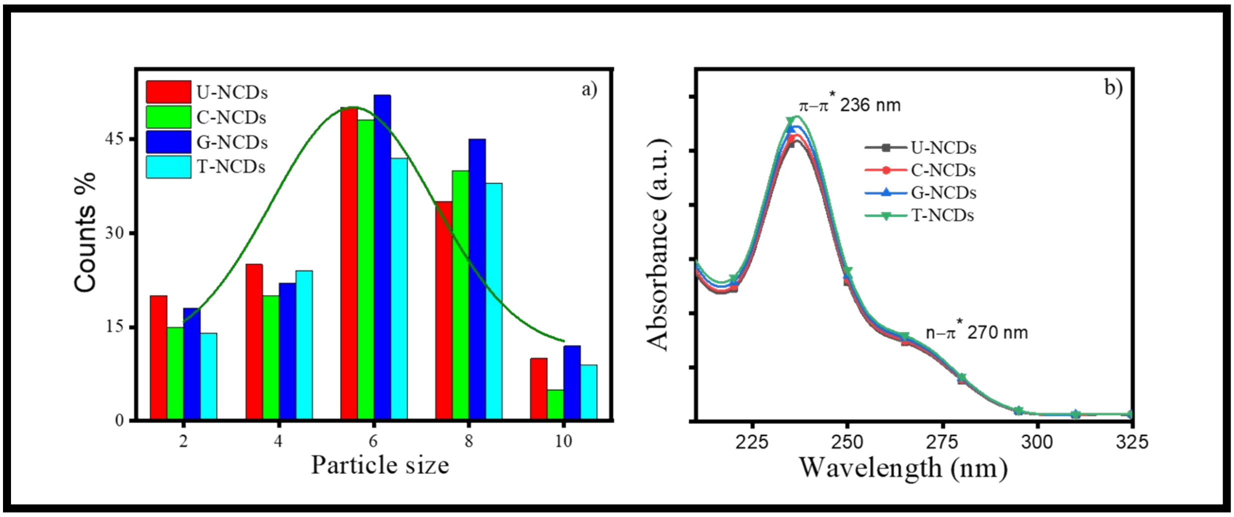

Fig. S1 Particle size distribution and UV-visible spectra of different NCDs

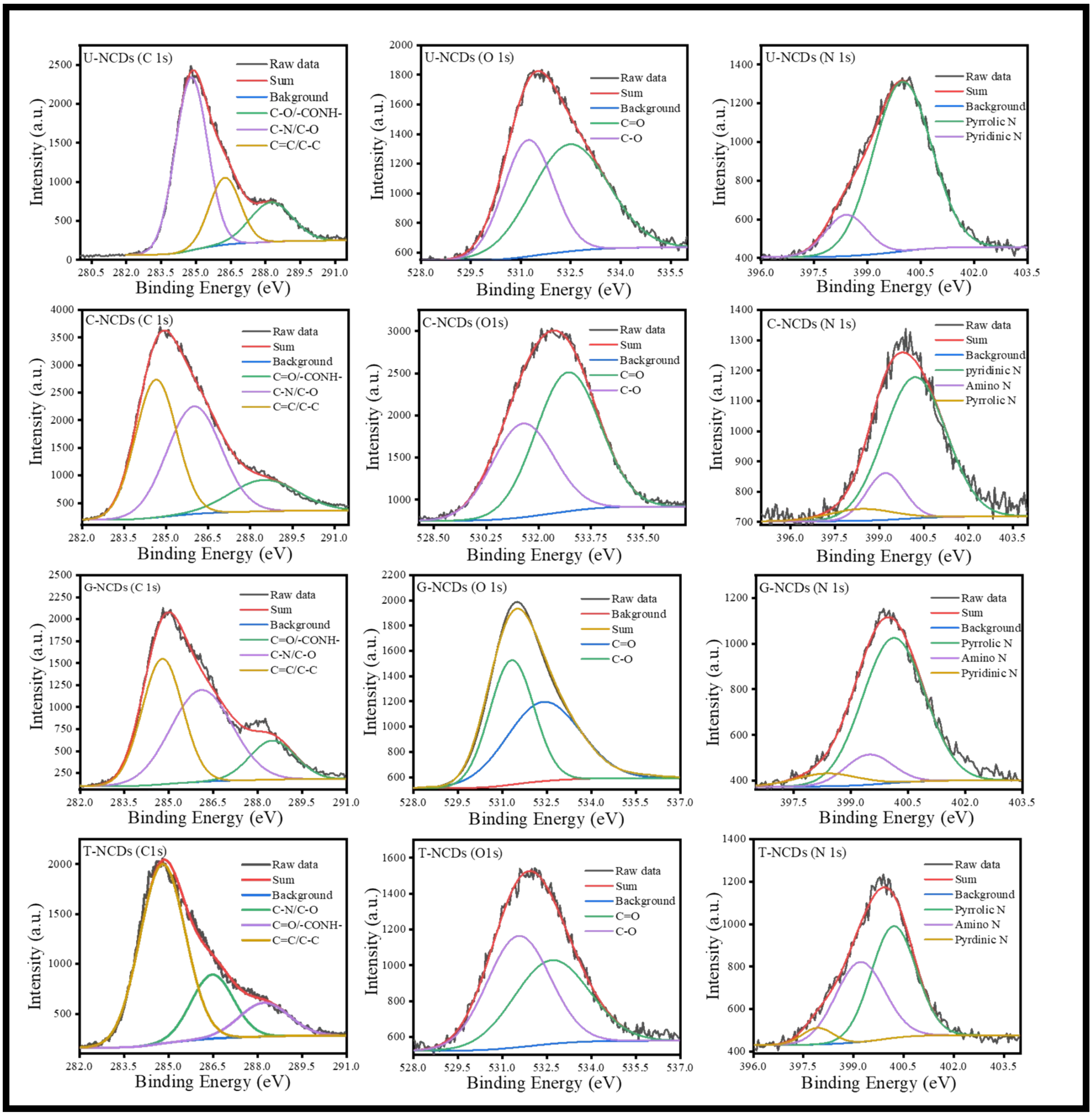

Fig. S2 Deconvoluted XPS spectra of C 1s O 1s and N 1s for: a) U-NCDs, b) C-NCDs, c) G-NCDs, and d) T-NCDs.

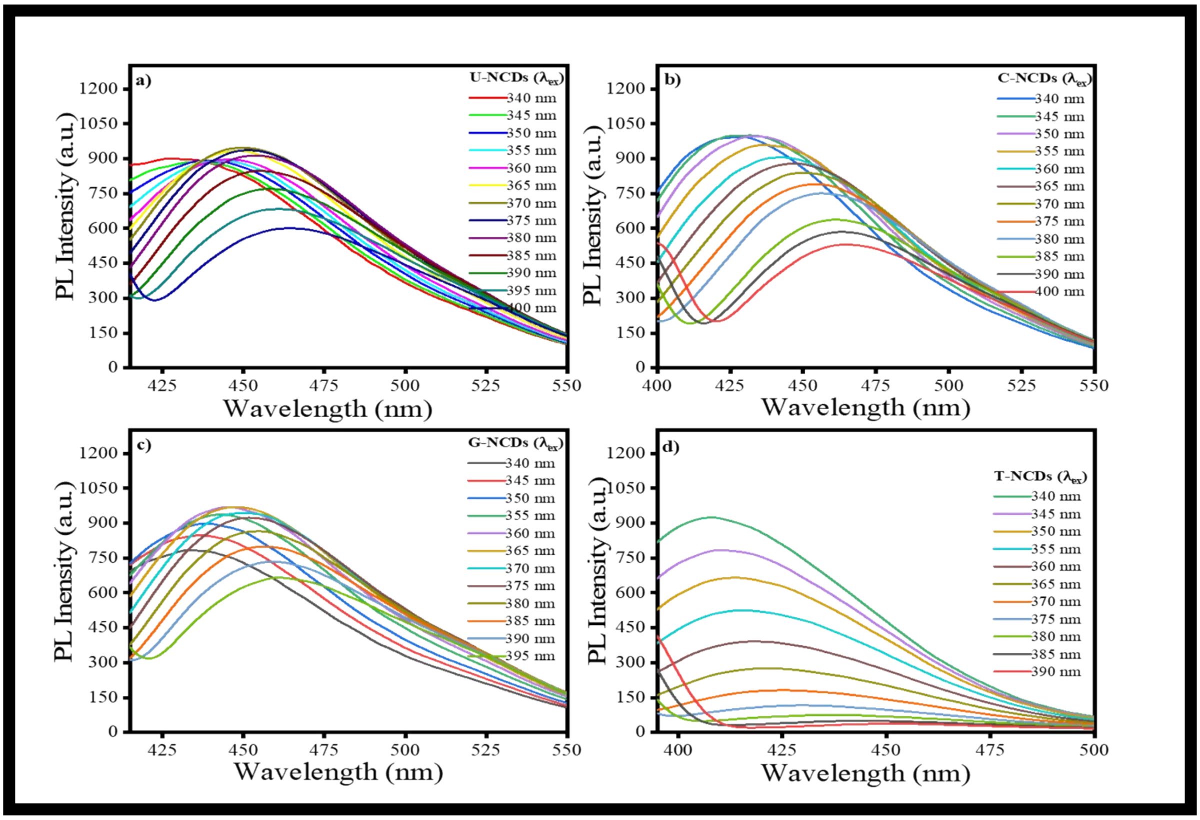

Fig. S3 Effect of excitation wavelength on emission spectra of:a) U-NCDs, b) C-NCDs, c) G-NCDs, and d) T-NCDs.

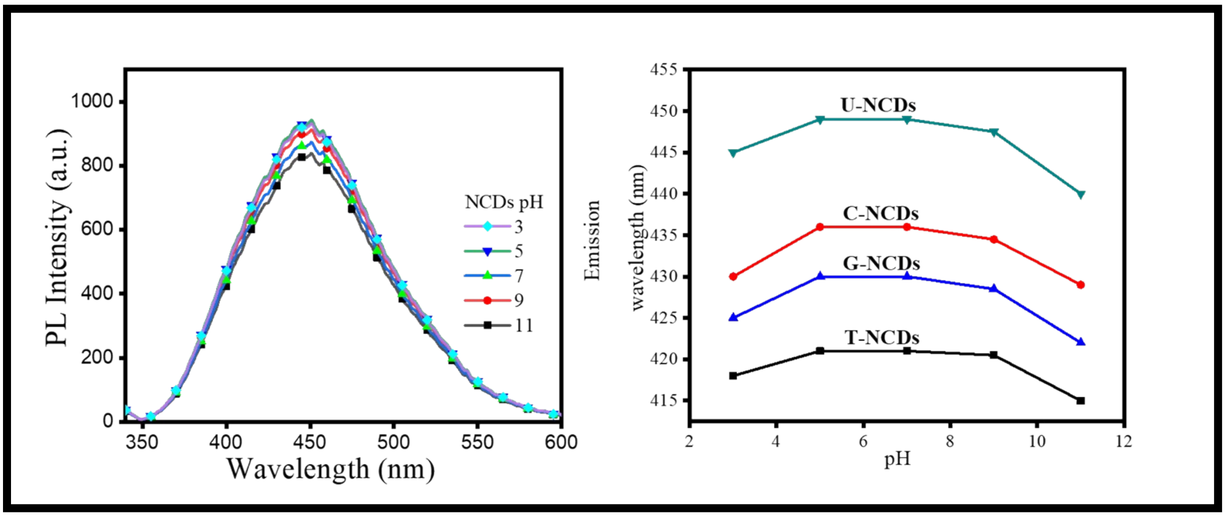

Fig. S4 Effect of pH a) PL intensity and b) emission wavelength of NCDs.

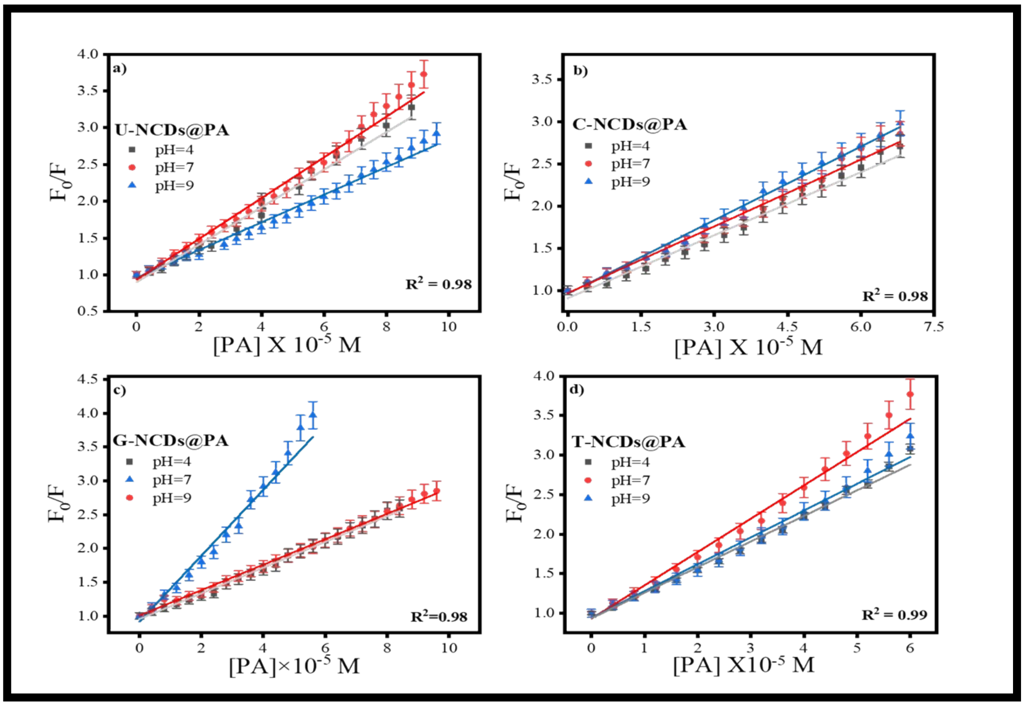

Fig. S5 Stern Volmer plot for a) U-NCDs b) C-NCDs c) G-NCDs and d) T-NCDs at different pH=4, 7 and 9 in the presence of PA.

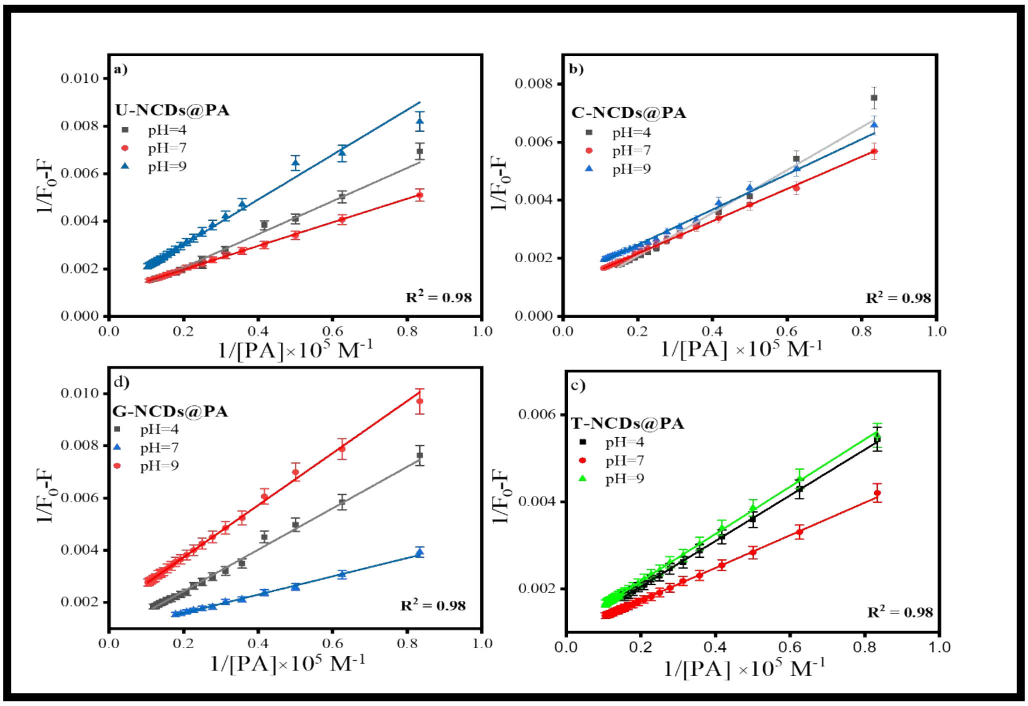

Fig. S6 B-H plot to evaluate the binding constant at pH=4, 7 and 9 for a) U-NCDs b) C-NCDs c) G-NCDs and d) T-NCDs in the presence of PA.

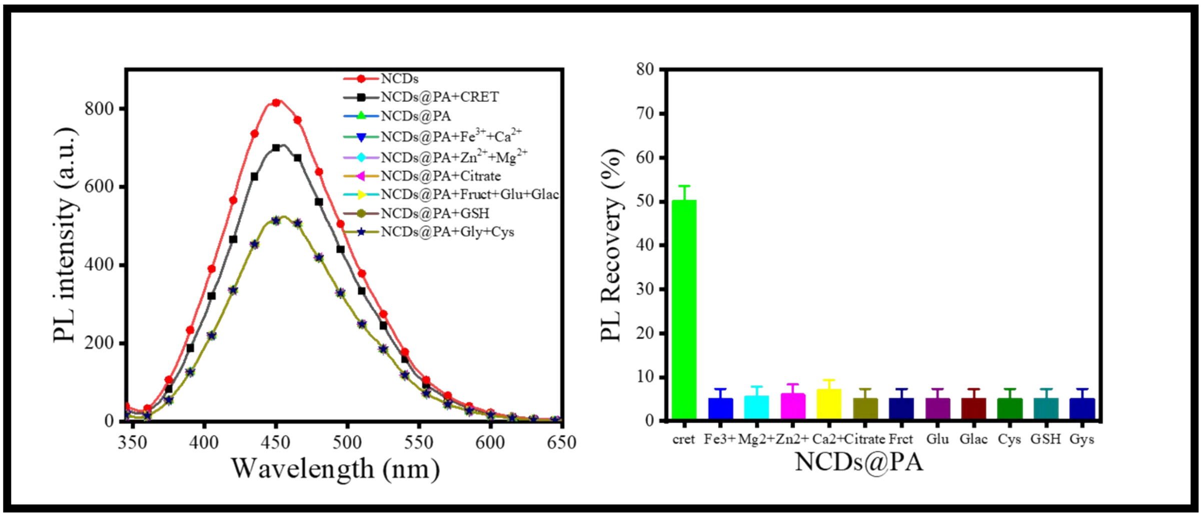

Fig. S7 Interference studies of NCDs@PA to evaluate the sensing efficacy towards creatinine.

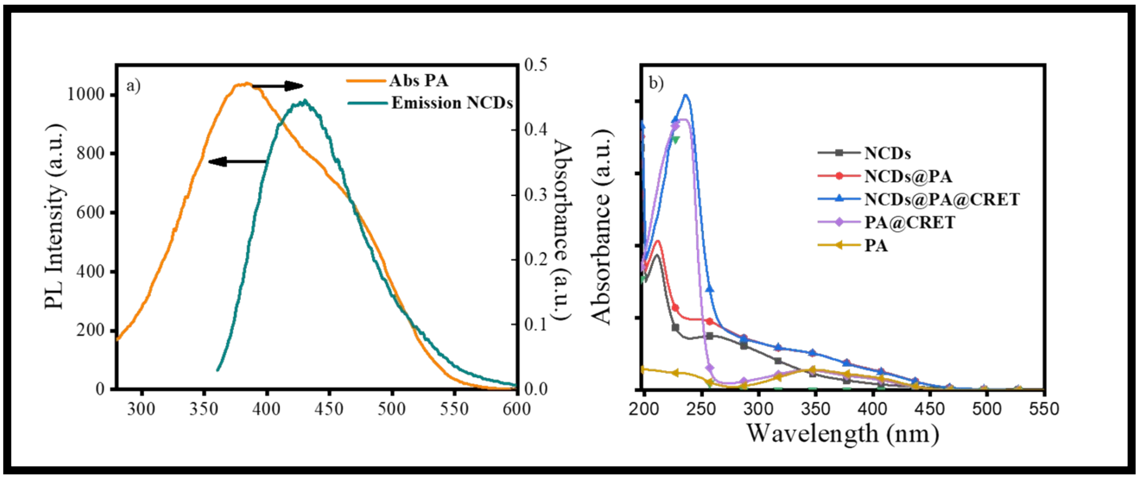

Fig. S8 Optical properties of NCDs a) combine absorption and emission spectra of PA and NCDs b) absorption spectra of NCDs, NCDs@PA and NCDs@PA@CRET.
